# A case report of seizure during emergence from general anesthesia after lumbar spinal surgery—common cases can develop potentially life-threatening adverse intracranial events

**DOI:** 10.1186/s40981-018-0179-9

**Published:** 2018-05-25

**Authors:** Junko Matsuhiro, Rumi Kariyazono, Koh Mizutani, Akinori Hinotsume, Masahiko Tsuchiya

**Affiliations:** 1 Department of Anesthesia, Osaka Rosai Hospital, 1179-3, Nagasonecho, Kita-ku, Sakai, 591-8025 Japan; 2 Department of Anesthesia, Osaka City General Hospital, 2-13-22, Miyakojimahondori, Miyakojima-ku, Osaka, 534-0021 Japan; 3 Department of Anesthesiology, Osaka City University Medical School, 1-5-7 Asahi-machi, Abeno-ku, Osaka, 545-8586 Japan

**Keywords:** Seizure, Emergence from general anesthesia, Subarachnoid hemorrhage, Intracranial hypotension, Spinal surgery, Posterior lumbar interbody fusion

## Abstract

**Background:**

Adverse intracranial events after spinal surgery were related with intracranial hypotension due to surgical injury of dura mater.

**Case presentation:**

A 72-year-old woman received posterior lumbar interbody fusion under general anesthesia. Immediately after the patient was transitioned to the supine position and muscular relaxants were reversed, she developed generalized seizure. The seizure was immediately suppressed with propofol. Brain computed tomography was unremarkable. Although she returned to the surgical suite, an evident point of dural laceration was not found. The dura was covered with fibrin glue. Magnetic resonance imaging revealed subarachnoid hemorrhage (SAH) on postoperative day 1. By postoperative day 2, the seizure had resolved. The cause of her seizure was suspected to be SAH due to intracranial hypotension. Seizure was masked by ongoing anesthesia and muscle relaxation.

**Conclusions:**

Although spinal surgeries are common procedure, we must carefully consider its related potentially life-threatening adverse events.

## Background

There have been numerous publications addressing adverse intracranial events after spinal surgery. Most of those events were related with intracranial hypotension due to surgical injury of dura mater. Sometimes, seizure appears as the manifestation of intracranial events [1–4]. In the previous reports, seizure appeared a few hours or days after the surgeries. Here, we report a case of seizure during emergence from general anesthesia. To the best of our knowledge, this is the first report to describe seizure during emergence from general anesthesia due to intracranial hypotension after spinal surgery.

## Case presentation

**Fig. 1:**
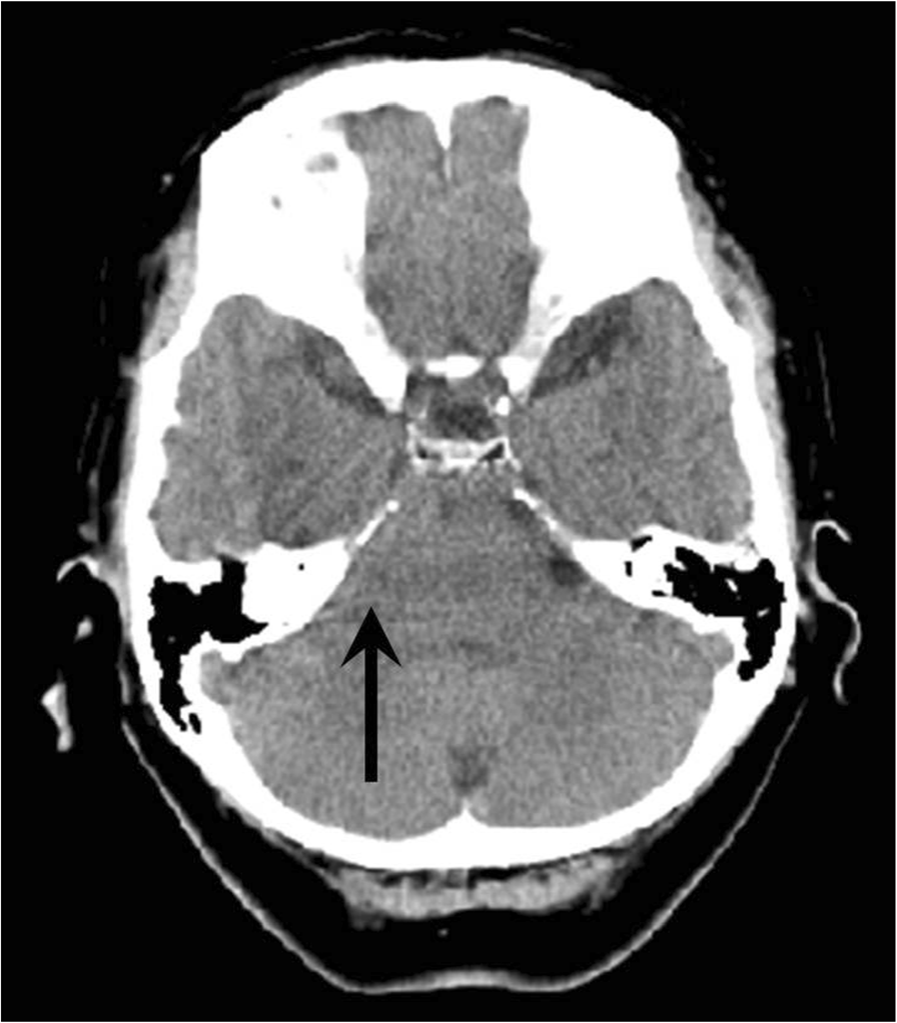
Post-surgical non-contrast computed tomography of the brain showing no intracranial hemorrhage. Subsequent image reading demonstrated the disappearance of the cerebrospinal fluid cavity at the right cerebellopontine angle (arrow)

A 72-year-old woman (140 cm, 56.9 kg) complained of low back pain and numbness and was admitted to our institution for posterior lumbar interbody fusion (PLIF) at L5-S1 and partial laminectomy at L2-L4. She had a prior history of PLIF at L4-5 at 68 years of age. She had no significant medical history except hypertension but no history of epilepsy. Her preoperative laboratory tests were within normal limits. Anesthesia was induced with propofol 70 mg and remifentanil 0.3 μg/kg/min. The trachea was intubated with an aid of rocuronium 50 mg. Anesthesia was maintained with desflurane 3–4% and remifentanil 0.1–0.2 μg/kg/min. Fentanyl 300 μg and rocuronium 20 mg were added during surgery. Bispectral index (BIS) was not monitored. Cerebrospinal fluid (CSF) leakage was not detected, even when inflation of the lungs by employing sustained positive end-expiratory pressure of 20 cm H_2_O for 5 s. The intraoperative course was uneventful and her hemodynamics remained stable during the 4 h and 55 min surgery. A 5-mm-diameter subfascial drainage tube was placed on the dorsal side of the lamina and opened about 10 min before the surgery was completed. After she was moved to the supine position, all anesthetics were stopped and the muscular relaxant was reversed with sugammadex 100 mg. Immediately thereafter, she developed symmetric, generalized, tonic seizure beginning in the upper extremities and advancing to the lower extremities. The seizure was immediately suppressed with propofol 80 mg; however, the cause of the seizure remained unclear. In order to rule out intracranial hemorrhage, brain computed tomography (CT) was performed under sedation with midazolam 10 mg. Brain CT showed no hemorrhage (Fig. 1). Arterial blood gas analysis showed normal acid-base balance, serum electrolytes, and glucose. Propofol was continued because of subsequent repeated seizure.

Although there was minimal output from the drainage tube, our surgical team suspected cauda equina incarceration or the other unknown cause of seizure in the surgical field, because they knew that any neurologic manifestation could appear by intracranial hypotension after spinal surgeries [5, 6]. Six hours after the initial surgery, the patient was taken back to the surgical suite to confirm and repair a suspected spinal dural laceration. The re-opened surgical field was wet with diluted blood. We suspected that the blood was diluted with the leaked CSF, because the fluid over there might not have the others of the CSF. However, an evident point of dural laceration was not found. There was no cauda equina incarceration. The dura was covered with fibrin glue.

**Fig. 2:**
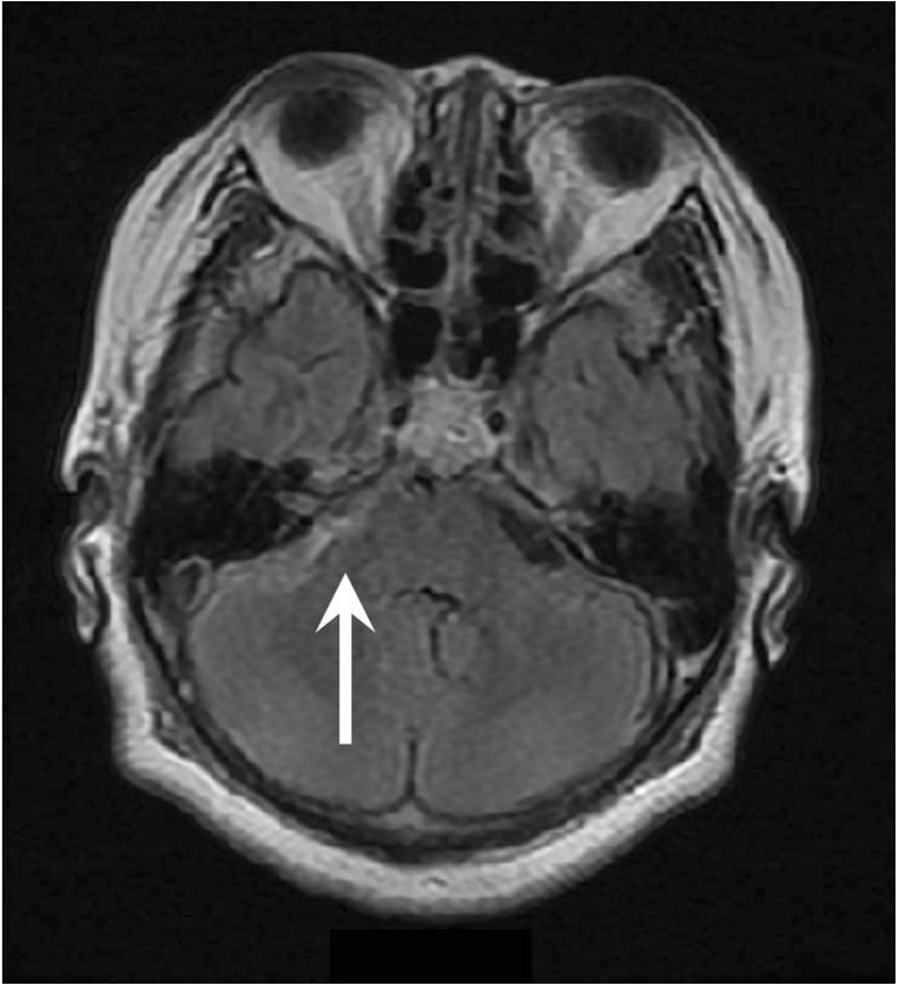
T2-weighted magnetic resonance imaging of the brain showing a subarachnoid hemorrhage at the right cerebellopontine angle (arrow)

On the first postoperative day, generalized seizure reappeared when sedation was held. Valproic acid 800 mg and levetiracetam 500 mg were added for anticonvulsive therapy. Brain magnetic resonance imaging (MRI) revealed a subarachnoid hemorrhage (SAH) at the right cerebellopontine angle (Fig. 2). At that time, we retrospectively noticed that a CSF cavity on the post-surgical brain CT had not been present (Fig. 1), suggesting intracranial hypotension. Ultimately, the cause of the seizure was thought to be SAH due to intracranial hypotension. The seizure resolved on postoperative day 2, and her trachea was extubated. She suffered severe headache with typical signs of a classic post-dual puncture headache. Therefore, she was maintained in the Trendelenburg position until postoperative day 3. She was discharged on postoperative day 27 without any neurologic sequelae, and valproic acid and levetiracetam were continued for an additional 3 weeks.

## Discussion

The majority of severe complications following spinal surgery are related to dural injury and intracranial hypotension [6]. The incidence of dural injury during spinal surgery has been reported as 5.5 to 10.1% [5, 7]. Dural injuries can cause intracranial hemorrhage [8, 9] and even stroke and death [10]. Seizure is the one of the manifestation of intracranial events [1–4]. In the previous reports, seizure appeared a few hours or days after the surgeries. However, seizure appeared during emergence from anesthesia in our patient, probably because larger dose of CSF spilled over from a subarachnoid space during surgery. When intraoperative dural injury is recognized, it is immediately repaired, preventing the subsequent development of postoperative intracranial hypotension. However, if the dural injury is not obvious during surgery like as our patient, the resultant CSF leakage can consequently lead to severe intracranial hypotension. Furthermore, the drainage tube can contribute to intracranial hypotension [7].

In retrospect, the actual onset time was thought when the drainage tube was opened and started suction. Seizure was masked by ongoing anesthesia and muscle relaxation; we recognized the seizure just after discontinuing anesthetics and reversal of the muscle relaxant. Regrettably, BIS was not monitored. If it had been monitored, abnormal spike waves might have been captured from the onset of the seizure. Although seizure activity might have damaged the central nerve system for a few minutes, it was fortunate that any neurologic sequelae had not been left. In order to detect earlier sign of intracranial hypotension that can lead to SAH and other catastrophic events, we should have knowledge of surgical procedures and their related potentially life-threatening adverse intracranial events even in common spinal cases. In addition, careful vigilance of patients and potentially useful BIS monitor are crucial. Brain CT and/or MRI are decisive to confirm intracranial hypotension and hemorrhage.

While the exact relation between intracranial hypotension and SAH is unclear in this patient, it has been postulated that extensive CSF loss can lead to a downward displacement of the brain and stretching/compromise of the bridging veins [11, 12]. In addition, an increased pressure gradient between venous blood and CSF can contribute to SAH [11, 12].

It is possible that the patient’s seizure stopped when the natural production of CSF resolved her intracranial hypotension. This is supported by the finding that plasma concentrations of valproic acid and levetiracetam had not reached therapeutic levels by postoperative day 2, and the patient’s headache disappeared on postoperative day 3. Headaches due to CSF leak are usually benign and 80% resolve within 5 days [12–14].

Symptomatic seizure after spinal anesthesia [15, 16], combined spinal-epidual anesthesia [17], and accidental dural puncture during epidural anesthesia [18, 19] have previously been reported. In these cases, intracranial hemorrhage after spinal and epidural anesthesia led to seizure. The pathophysiological mechanisms causing intracranial hypotension after spinal and epidural anesthesia are likely to be the same as those caused by spinal surgeries.

In the case of seizure during emergence from general anesthesia, we had to diagnose the cause of seizure immediately and manage it correctly. Causes of seizure following general anesthesia include epilepsy, hyponatremia, hypocalcemia, hypoglycemia, hyperglycemia, uremia, hepatic encephalopathy, local anesthetic toxicity, intracranial lesions, antibiotic solution used to irrigate the wound, and foreign matter in the CSF cavity. Our patient had no history of epilepsy. Arterial blood gas analysis, blood tests, and electroencephalography were normal. Initial brain imaging was unremarkable. Also, no antibiotic solution was used to irrigate the wound before wound closure. Hence, intracranial hypotension was not initially considered as a cause of her seizure.

## Conclusions

It is important for anesthesiologists to carefully understand the surgical procedure and its related potentially life-threatening adverse intracranial events even in common cases, whenever encountering unforeseen events in the perioperative period.

## Abbreviations

CSF: Cerebrospinal fluid
CT: Computed tomography
MRI: Magnetic resonance imaging
PLIF: Posterior lumbar interbody fusion
SAH: Subarachnoid hemorrhage
